# Remote detection of water stress in cotton using a center pivot irrigation system-mounted sensor package

**DOI:** 10.1038/s41598-024-74092-2

**Published:** 2024-10-08

**Authors:** Bala R. Sapkota, Curtis B. Adams, Qiong Su, Srinivasulu Ale

**Affiliations:** 1 Department of Soil and Crop Sciences, Texas A&M University, 370 Olsen Blvd, College Station, TX 77843 USA; 2 USDA Agricultural Research Service, Columbia Plateau Conservation Research Center, 48037 Tubbs Ranch Road, Adams, OR 97810 USA; 3 Department of Agricultural Sciences, Clemson University, 232 McAdams Hall, Clemson, SC 29634 USA; 4 Texas A&M AgriLife Research, , 11708 Hwy. 70 South, Vernon, TX 76384 USA

**Keywords:** Crop water stress index, Water deficit index, Infrared temperature sensor, Normalized difference vegetation index, DSSAT, Unmanned aircraft systems, Plant sciences, Engineering

## Abstract

Much research has been invested in infrared temperature (IRT)-based methods for cotton (*Gossypium hirsutism* L.) water stress detection using in-field sensors, but adoption of these is low, perhaps due to logistical challenges. Alternatively, the Water Deficit Index (WDI) was developed for crop water stress assessment using remote sensors not embedded in the canopy. The objective of this research was to evaluate the performance of a sensor package—including modern IRT and normalized difference vegetation index (NDVI) sensors facing downward at 45˚, and a mini weather station—attached unintrusively to a center pivot irrigation system for detecting cotton water stress using WDI. Sensor packages were evaluated in a two-year field study that included four irrigation treatments (0, 30, 60, and 90% ET replacement) and in two production cotton fields. Overall, the tested system was effective at distinguishing crop water stress among irrigation rates. Comparison of the results to a ground-based station and simulations indicated that WDI overestimated water stress at the highest irrigation rate, but performed well otherwise. Accuracy of the system could be improved by measuring canopy coverage (*Fc*) from the same vantage point as the IRT and NDVI sensors (from the pivot, downward at a 45˚ angle).

**Supplementary information:**

The online version contains supplementary material available at 10.1038/s41598-024-74092-2.

## Introduction

According to climate model projections, hotter temperatures and more severe droughts are expected in the American Southwest^1,2^. Since water availability is a primary factor determining plant productivity, the changing climate will become an increasingly important issue for crop producers. Upland cotton is popularly grown, for example, in most regions of Texas and the surrounding region. An average of 32% of the area planted to cotton in Texas was irrigated in 2018, but that percentage varied from 8% in the Rolling Plains to about 55% in the Northern High Plains^3^. However, approaches to cotton water management are shifting in response to a variety of factors. Irrigated cotton typically produces more lint with better quality fiber than dryland production, making irrigation a desirable choice for producers if water is available^4,5^. Recurring droughts and extreme weather are making dryland cotton yields more unpredictable, increasing rates of evapotranspiration (ET) and the demand for irrigation water^6^. But the primary source of irrigation water in the state, groundwater, is also being rapidly depleted by elevated demand for irrigation that far exceeds recharge from rainfall. For example, water levels in the Seymour Aquifer in the Texas Rolling Plains and Ogallala Aquifer in the Texas High Plains region are being drawn down due to overuse^7–9^. This has substantially reduced the irrigation well capacities, especially in the High Plains^6,7^.

In the cotton-growing regions of Texas, drip and sprinkler irrigation systems have largely supplanted the less efficient flood irrigations systems^10–12^. Overhead sprinkler irrigation systems (e.g., center pivots and linear systems) have been increasingly adopted because they are quick to assemble, durable, relatively easy to operate, and adjustable^12^. However, efficient irrigation systems, like pivots and linears, can also result in negative consequences, if executed poorly. In addition to the challenge of depleting aquifers, excessive irrigation of cotton can induce seedling diseases^13^, increase pest infestation^14^, and induce leaching of nutrients^15^, which can negatively impact yield and environmental quality^16^. Compared to full ET replacement through irrigation, studies have shown that full yield potential can be achieved with use of less water by adopting more efficient irrigation management strategies^15,17,18^. Thus, an overarching goal in modern irrigation management is to simultaneously optimize yield and irrigation water-use efficiency, which is only possible through better understanding of crop water stress dynamics and effective scheduling of irrigation.

Current approaches to sprinkler irrigation scheduling often include monitoring soil or plant water status, then irrigating at predetermined thresholds. Some examples include monitoring soil water potential with tensiometers, measuring soil moisture by electronic sensors, tracking leaf-water potential by various means, calculating ET replacement using meteorological data and crop coefficients, and calculating a crop water stress index (CWSI) using canopy temperature and other meteorological variables^19,20^. Each of these irrigation management and scheduling approaches have some benefits and drawbacks. Soil moisture-based methods, for example, require in-field installation of sensors that can pose logistical challenges for field operations and have severe spatial limitations, even if resources are invested to install them extensively. Most CWSI-type approaches also rely on in-field infrared temperature (IRT) sensors that have these same challenges and limitations. Some sensor-based systems, such as thermal imagers, currently have high cost and complex post-processing requirements that make them inaccessible to most crop producers. One possible solution to these practical problems in center pivot and linear irrigation systems is to mount IRT and normalized difference vegetation index (NDVI) sensors on the irrigation systems and use them in conjunction with meteorological data to implement sensor-based irrigation monitoring and control on the go. This approach would eliminate in-field deployment of sensors and allow crop water stress data to be collected right where the pivot is irrigating as it moves around the field.

Canopy temperature has been long recognized as a good indicator of crop water stress and CWSI is one of the primary crop water status models used for irrigation scheduling^21,22^. The CWSI model requires the calculation of three types of canopy-to-air temperature differences, including the actual canopy-to-air temperature difference (i.e., Tc-Ta), the canopy-to-air temperature difference for a well-watered crop surface with full transpiration, and the canopy-to-air temperature difference for a dry crop surface with no transpiration^21–27^. Canopy temperature is measured by non-contact IRT or thermal imagers. However, until full canopy cover is reached, canopy temperature measurements from IRTs placed above the canopy are influenced by soil temperature within the field of view of the sensor. To overcome this challenge and limitation, Moran et al.^28^ modified the CWSI model into the Water Deficit Index (WDI) by introducing a crop cover fraction (*Fc*) parameter that addressed the influence of soil background at partial vegetation cover.

Colaizzi et al.^25^ demonstrated that the WDI determined using a pivot-mounted sensor system (4 m height aboveground), called “The Agricultural Irrigation Imaging System,” or AgIIS, could detect water stress in cotton. The system included a thermal sensor to record canopy temperature, a spectroradiometer with reflectance bands to estimate NDVI, and *Fc* was manually measured by destructive sampling to establish a relationship with NDVI. The system relied on an on-site weather station to collect needed meteorological parameters. They also used capacitance probes to record change in volumetric soil moisture content. The investigators did not find a good relationship between soil water depletion and WDI. However, good correlation (R^2^ = 0.84 to 0.87) was observed between the WDI and the soil water deficit index (SWDI), which is based on ET and a crop water stress coefficient (Ks). Since that report was published, relatively inexpensive NDVI sensors are now commercially available that can continuously measure NDVI in field conditions^29,30^. There have also been recent advances in development of commercially available all-in-one, compact weather stations that can be economically deployed anywhere^31^. On-site standard weather stations are usually available in research settings, but the source of this data will typically be farther away from actual producer fields where such systems are intended to be utilized.

Crop producers need relatively simple and accessible technologies to detect crop water stress and improve irrigation management. Use of a WDI-based sensor package mounted unintrusively on center pivot or linear irrigation systems has been demonstrated to successfully predict soil moisture status^25^. However, additional research is needed to develop such a technology for use by producers, which includes testing the package with modern sensors that can be easily and economically deployed in production settings. Thus, the first objective of this research was to test a pivot-mounted sensing package with modern IRT and NDVI sensors, plus an integrated mini weather station, to detect crop water stress in cotton via the WDI model. The second objective was to compare the results to simulated results using a crop growth model, and then determine the viability of generating irrigation prescriptions based on the simulation model output.

## Results

### NDVI and Fc

**Fig. 1:**
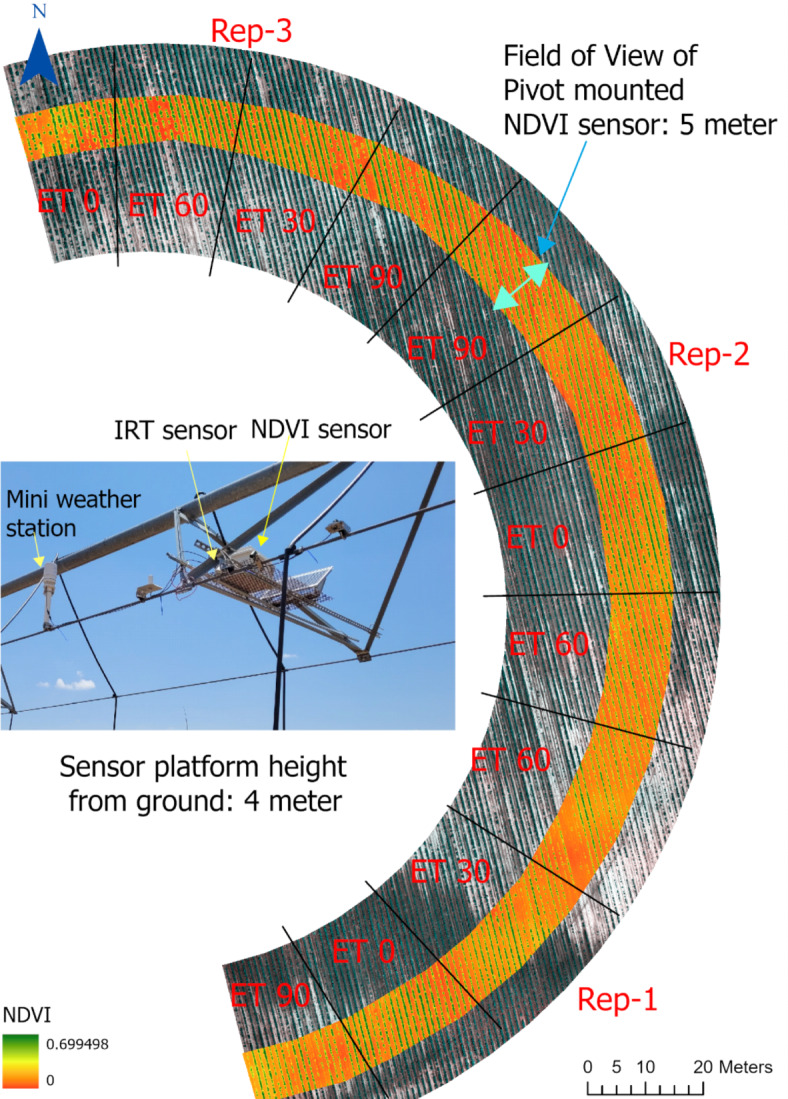
An image of a pivot-mounted sensor platform (inside) and an image of the field from the two-year study at the Chillicothe Research Station (outside). In the field image, plot lines have been approximately drawn and the area within the field of view of sensor is designated by the orange strip.

**Fig. 2:**
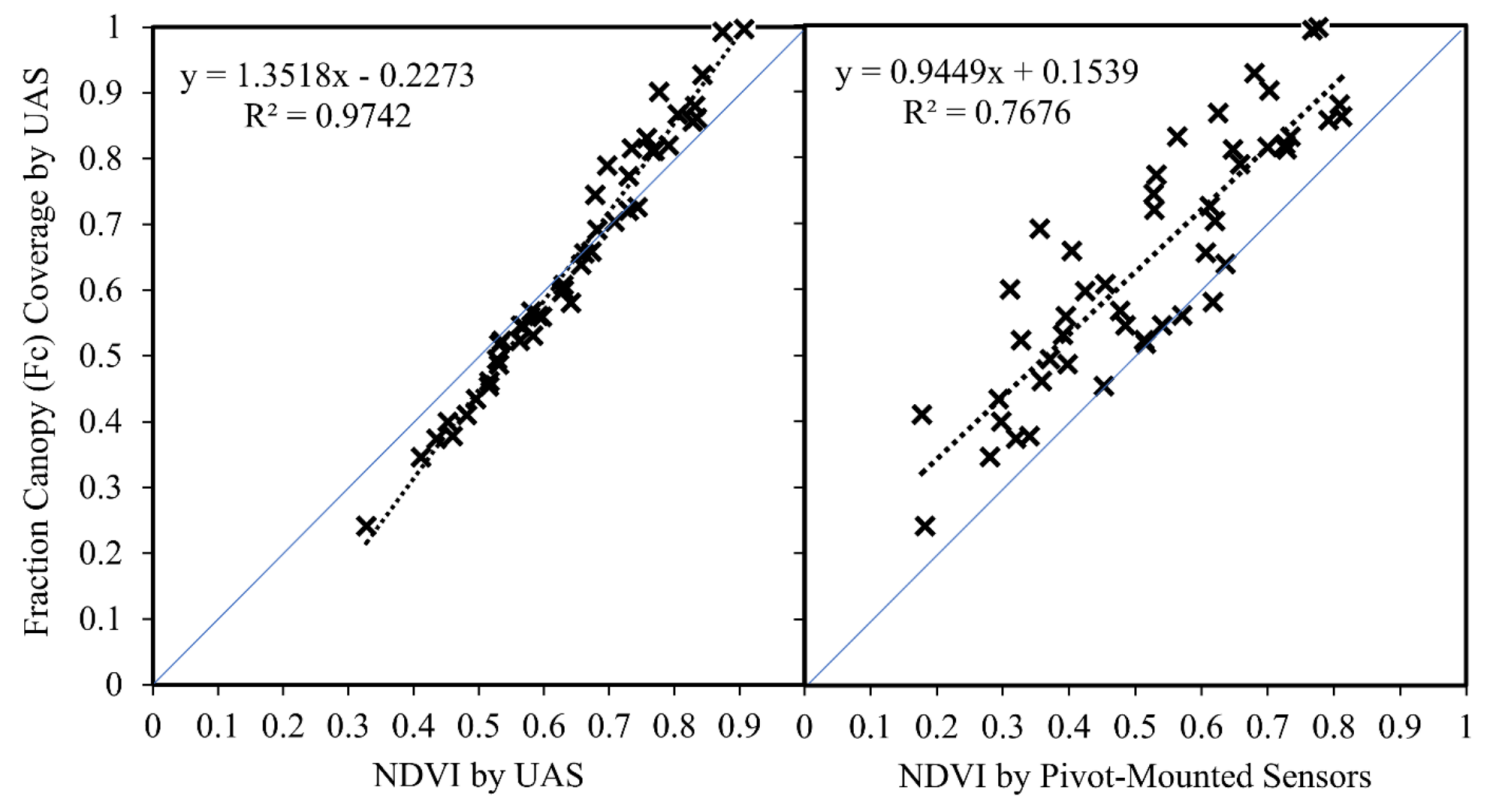
Relationships between NDVI and fraction canopy cover (*Fc*) in 2021 at the Chillicothe Research Station. On the left, NDVI was measured by UAS. On the right, NDVI was measured by pivot-mounted NDVI sensor. *Fc* was measured by UAS in both cases.

**Fig. 3:**
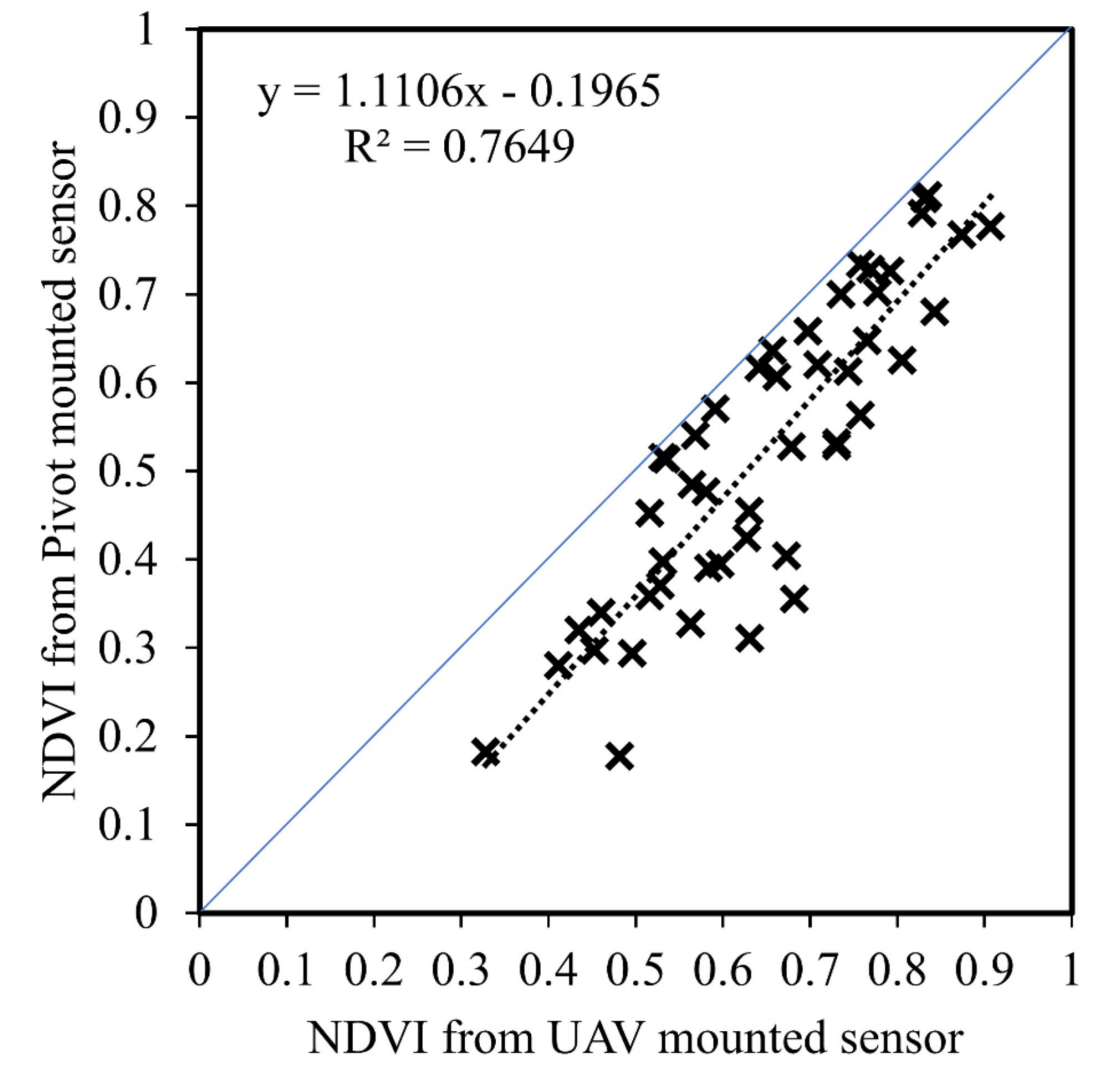
The relationship between the NDVI spectral index as measured by UAS and by pivot-mounted sensors.

When NDVI was measured by pivot-mounted sensor and *Fc* was measured by UAS at Chillicothe in 2021, there was a linear relationship between these parameters (Figs. 1, 2). There was less variability in the relationship when both parameters were derived by UAS (R^2^ = 0.97) and more variability when the sources were different (R^2^ = 0.77). The primary source of this variability was the NDVI output of the pivot-mounted sensor. The relationship between NDVI from both sources is shown in Fig. 3. There was also a difference in slope (m) between the two relationships: m = 0.94 from pivot NDVI vs. UAS *Fc* and m = 1.35 for UAS NDVI vs. UAS *Fc* (Fig. 2).

### Weather parameters

Comparisons were made between weather data collected by the mini weather stations, which were integrated into pivot sensor packages at three locations (the Chillicothe Research Station, Production Pivot 1, and Production Pivot 2), and a standard weather station at Chillicothe (Table 1). Comparisons were also made between the weather parameters collected by the pivot-mounted and ground-based mini weather stations at Chillicothe (Table 1). There was strong agreement among all weather stations in measurements of *Rn* (R^2^ = 0.98 – 0.99). There was somewhat more disagreement among weather stations in measurements of Ta and RH (R^2^ = 0.96 – 0.99 and 0.94 – 0.98, respectively), though agreement was still strong. There was no apparent trend in R^2^ values for Ta and RH consistent with the location or height of the sensors. There was greater variability in wind speed measurements with sensor location and height, with R^2^ values ranging overall from 0.7 – 0.9. Compared to the wind speed measurements by the standard weather station at Chillicothe, the ground-based station on the pivot had greater agreement than the pivot-mounted sensor (R^2^ = 0.87 vs. 0.70). For the production pivots, agreement of wind speed measurements with the standard weather station decreased somewhat with distance from the standard station (R^2^ = 0.78 vs. 0.72). Disagreement between producer pivot-mounted sensors and the standard weather station was greater for precipitation measurements than all other weather parameters, with variability increasing with increasing distance between stations (R^2^ = 0.46 – 1.0).

**Table 1:** Relationship metrics of meteorological variables that are essential to calculating the Water Deficit Index (WDI) as measured by different weather stations at different locations.

Variables	Number of observations	Source X	Source Y	R^2^
Solar radiation (MJ m^-1^ day^-1^)	215	^#^ CRS Pivot	* CRS pivot ground	0.99
	215	CRS Pivot	^+^ CRS weather station	0.99
	1405	CRS pivot ground	CRS weather station	0.99
	1809	^!^ Production pivot 1	CRS weather station	0.98
	1809	^^^ Production pivot 2	CRS weather station	0.99
Wind speed (m/s)	215	CRS pivot	CRS pivot ground	0.90
	215	CRS pivot	CRS weather station	0.70
	1405	CRS pivot ground	CRS weather station	0.87
	1809	Production pivot 1	CRS weather station	0.78
	1809	Production pivot 2	CRS weather station	0.72
Relative humidity (%)	215	CRS pivot	CRS pivot ground	0.97
	215	CRS pivot	CRS weather station	0.95
	1405	CRS pivot ground	CRS weather station	0.95
	1809	Production pivot 1	CRS weather station	0.96
	1809	Production pivot 2	CRS weather station	0.94
Air temperature (°C)	215	CRS pivot	CRS pivot ground	0.98
	215	CRS pivot	CRS weather station	0.96
	1405	CRS pivot ground	CRS weather station	0.99
	1809	Production pivot 1	CRS weather station	0.98
	1809	Production pivot 2	CRS weather station	0.97
Precipitation (mm)	215	CRS pivot	CRS pivot ground	1.00
	215	CRS pivot	CRS weather station	0.92
	1405	CRS pivot ground	CRS weather station	0.94
	1809	Production pivot 1	CRS weather station	0.85
	1809	Production pivot 2	CRS weather station	0.46

The “CRS Weather Station” at the Chillicothe Research Station is a standard weather station, while data was collected using mini weather stations in all other cases.

^#^ClimaVue 50 mini weather station mounted on the pivot at Chillicothe with sensors at 4 m height from ground.

*ClimaVue 50 mini weather station mounted on the ground in a 90% ET replacement plot at Chillicothe with sensors at 2 m height from ground.

^+^Standard weather station at the Chillicothe Research Station located about 500 m from the study field with sensors at 2 m height from ground.

^!^Production Pivot 1 was located about 1.5 km from the standard weather station at Chillicothe.

^^^Production Pivot 2 was located about 5 km from the standard weather station at Chillicothe.

### WDI estimates

**Fig. 4:**
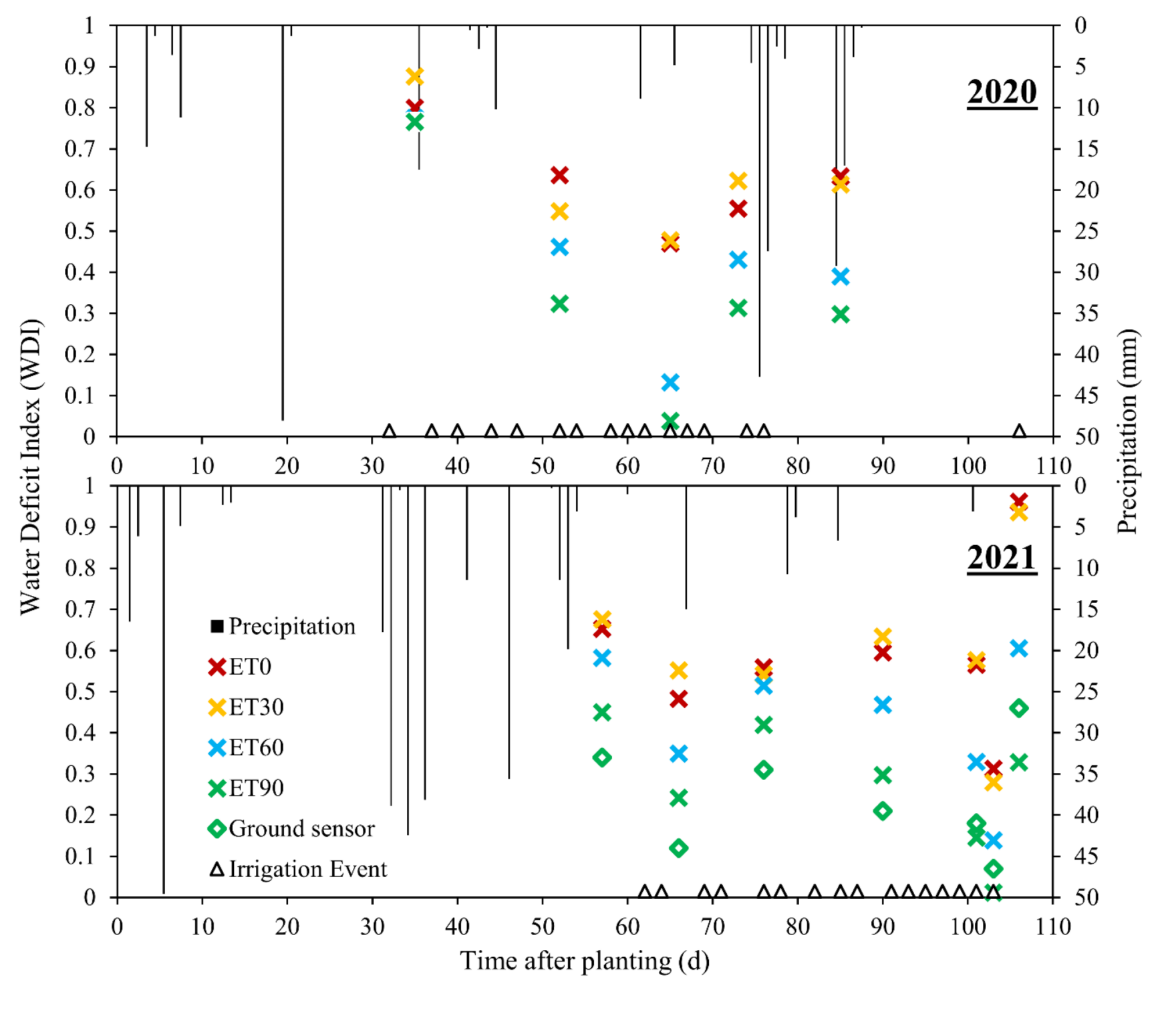
Water deficit index or WDI computed using data from a pivot irrigation system-mounted sensing package for different irrigation treatments at the Chillicothe Research Station in 2020 and 2021. ET90 refers to 90% ET replacement, ET60 refers to 60%, and so on. The “ground sensor” WDI data was derived from a sensing package with the temperature sensor focused directly on the crop canopy, which was placed in one ET90 plot.

**Fig. 5:**
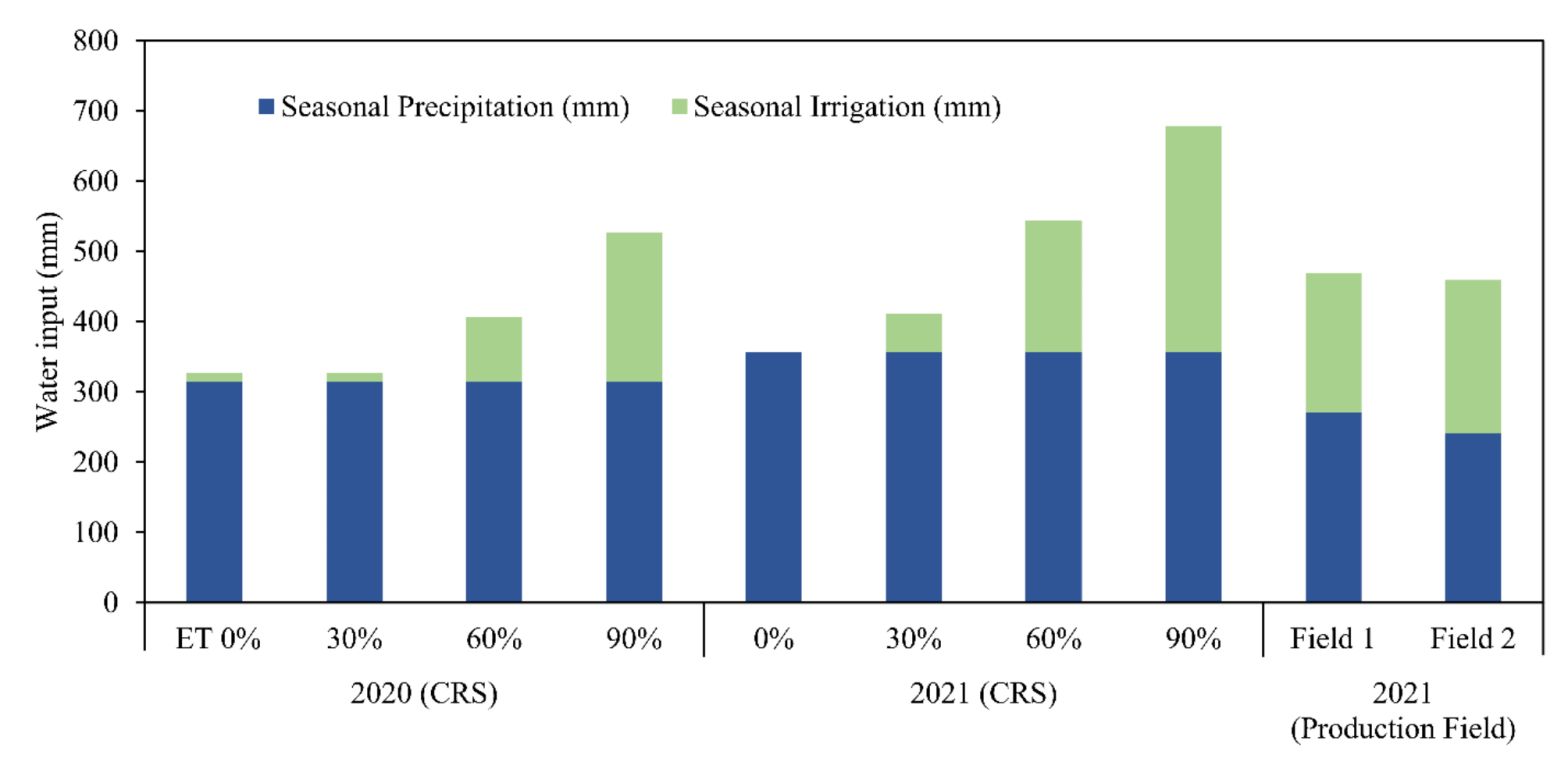
Total seasonal water input for cotton in 2020 and 2021 at the Chillicothe Research Station and on two production fields. Water was applied by pivot irrigation systems to replenish the percentage of water loss due to ET at different levels at Chillicothe. In the production fields, producer-chosen irrigation practices were followed and irrigation water was also applied by pivot systems.

There were differences in pivot sensor-derived WDI among ET replacement treatments at Chillicothe in both study years (Table 2; Fig. 4). The seasonal average value of WDI was the lowest (0.31—0.32) at 90% ET replacement, intermediate with 60% ET replacement (0.41—0.48), and the greatest with 30% and 0% (dryland) ET replacement (0.61 – 0.69) in both years (Table 2). The lack of difference in WDI between the 30% and 0% ET replacement treatments was due to minimal irrigation at 30%, because precipitation largely fulfilled water demands at that level (Fig. 5). Over the course of the season, the WDI values in each treatment shifted up and down somewhat (Fig. 4), reflecting plant responses to irrigation and precipitation events (Fig. 5). The WDI estimates from the pivot-mounted sensors at 90% ET replacement were higher than from the ground-based sensors that were placed in the same irrigation treatment until about 90 days after planting. Averaged over the season, WDI derived from the pivot-mounted sensor was about 19% higher than the ground-based WDI (0.31 vs 0.25). The relationship between the WDI calculated using pivot-mounted and ground based sensor measurement on a 90% ET replacement plot at Chillicothe in 2021 was R^2^ = 0.58.

**Table 2:** Statistical significance and summary of lint yield and seasonal average Water Deficit Index (WDI) for different irrigation treatments in the 2020 and 2021 seasons at the Chillicothe Research Station.

Treatments	Mean Lint Yield (kg ha^-1^)		Seasonal Avg. WDI	
	2020	2021	2020	2021
0% ET replacement	625b^+^	576c	0.60a	0.69a
30% ET replacement	471b	663c	0.61a	0.67a
60% ET replacement	739ab	1126b	0.42b	0.49b
90% ET replacement	1134a	1670a	0.32c	0.31c
*P*-values	*0.0125*	< *0.0001*	< *0.0001*	< *0.0001*

^+^Means within columns followed by the same letter are not significantly different according to P ≤ 0.05.

**Fig. 6:**
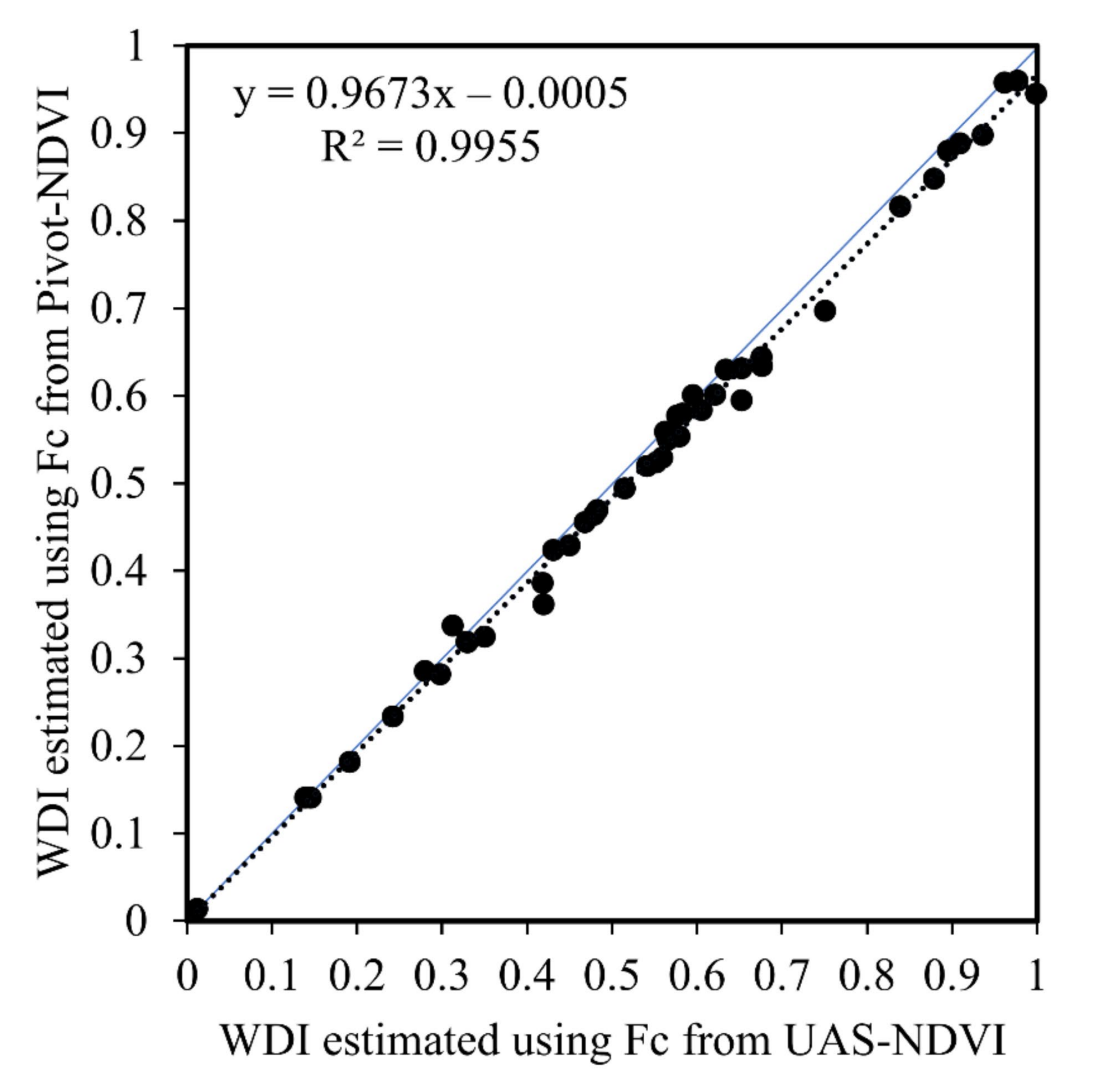
A comparison of WDI values calculated using both of the NDVI-*Fc* relationships shown in Fig. 2 [NDVI (pivot)-*Fc* (UAS) and NDVI (UAS)-*Fc* (UAS)].

As described earlier, the NDVI (pivot) vs. *Fc* (UAS) relationship had wide variability (Fig. 2). To understand how this variability may have affected WDI values calculated using this relationship, WDI as calculated using both of the NDVI-*Fc* relationships shown in Fig. 2 [i.e., NDVI (pivot)-*Fc* (UAS) and NDVI (UAS)-*Fc* (UAS)]. A graphic comparing WDI using these approaches is shown in Fig. 6. Little variability is evident, though there tended to be a slight departure from a 1:1 relationship as WDI increased. Unfortunately, no measurements of *Fc* were taken from the vantage point of the pivot-mounted NDVI or IRT sensors for comparison.

**Fig. 7:**
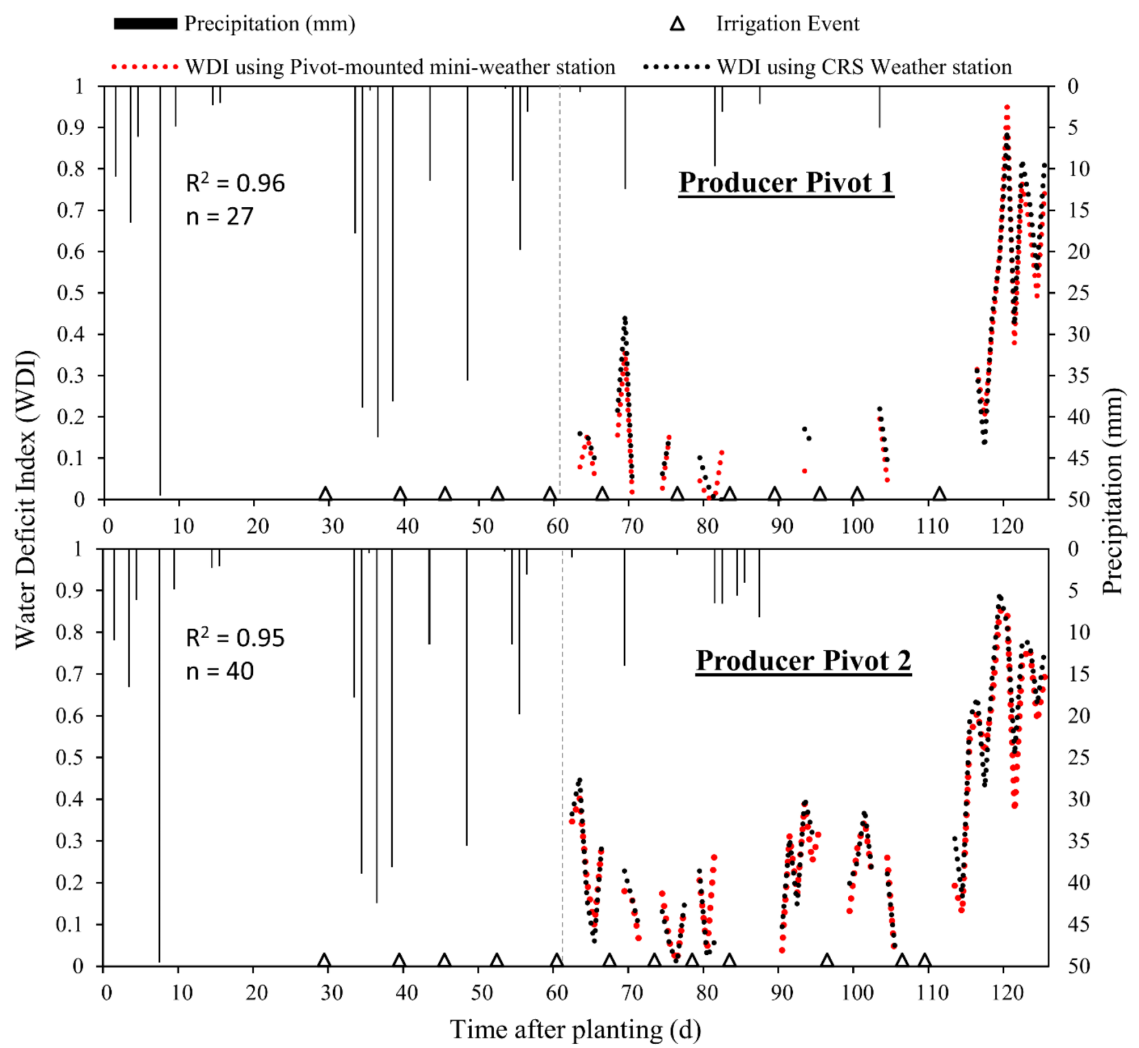
Water deficit index (WDI) computed for cotton on two production pivot irrigation systems in 2021. Sensor platforms were installed on the pivots at just more than 60 days after planting. Comparisons are made of WDI computed using data from system-integrated mini-weather stations and data from a distant standard weather station at the Chillicothe Research Station. Production Pivot 1 and Production Pivot 2 were located about 1.5 km and 5 km from the standard weather station, respectively. The precipitation data shown in both graphs before the vertical dashed lines is derived from the standard weather station, and data after the dashed line comes from the integrated mini-weather stations.

Similar to the data from Chillicothe, the WDI values from Production Pivots 1 and 2 shifted up and down over the course of the season, reflecting canopy temperature responses to irrigation and precipitation events (Fig. 7). Importantly, this data served to show that valid WDI data could be obtained from pivot-mounted sensors that were taking measurements while the pivot was actively applying irrigation water. The WDI derived from the production pivots using weather data from pivot-mounted mini weather sensors were compared with WDI using weather data from the standard weather station at Chillicothe, located 1.5 to 5 km away (Production Pivots 1 and 2, respectively). Variability in WDI increased only minorly with distance from the standard weather station: R^2^ = 0.96 for Production Pivot 1 and R^2^ = 0.95 for Production Pivot 2 (Fig. 7).

### Comparison of sensor-based WDI and DSSAT-simulated WDI at chillicothe

**Fig. 8:**
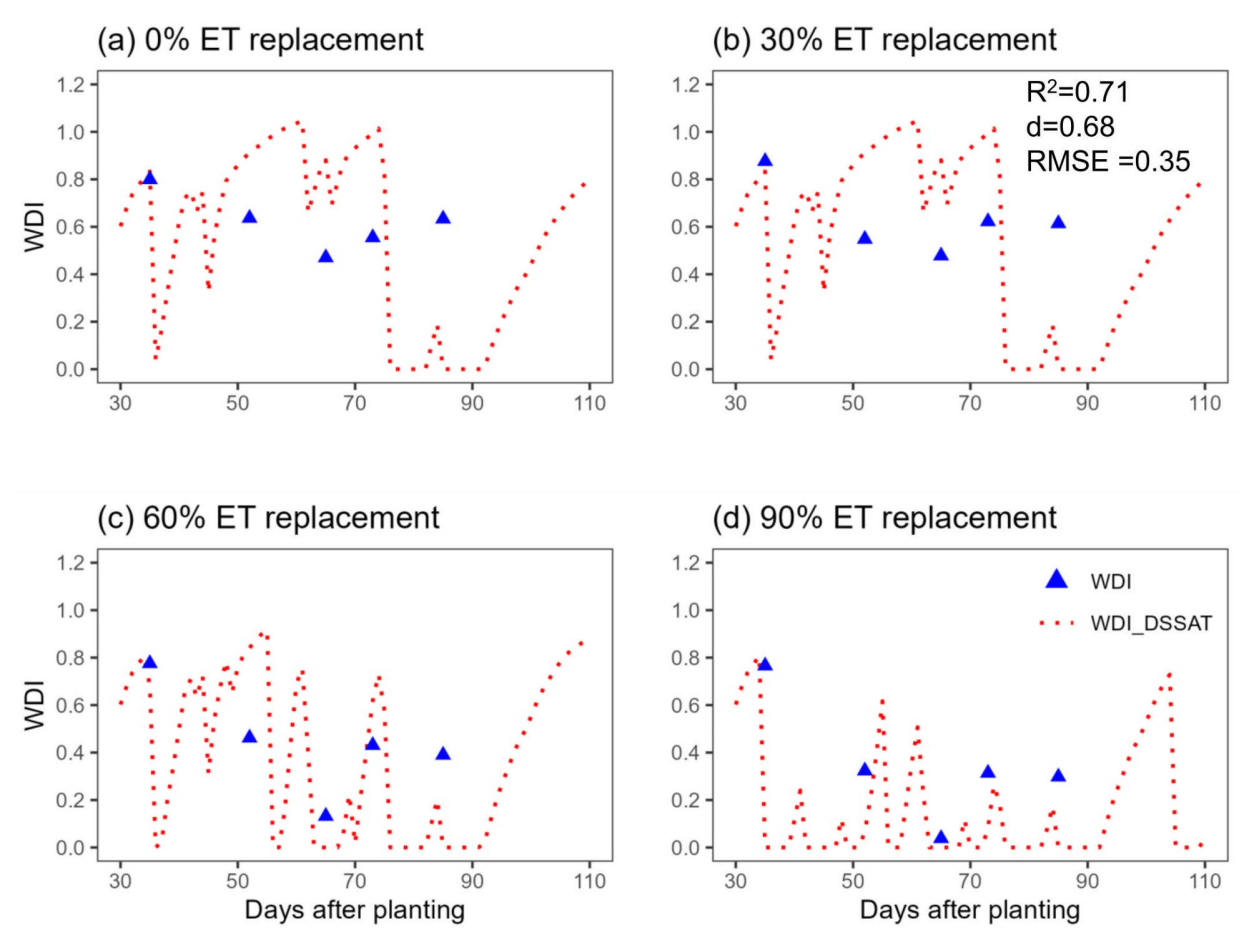
Comparison of Water Deficit Index (WDI) calculated directly using field data from a pivot-mounted sensor package and simulated using the Soil Water Balance method in DSSAT for cotton managed under different ET replacement irrigation regimes in the 2020 growing season.

**Fig. 9:**
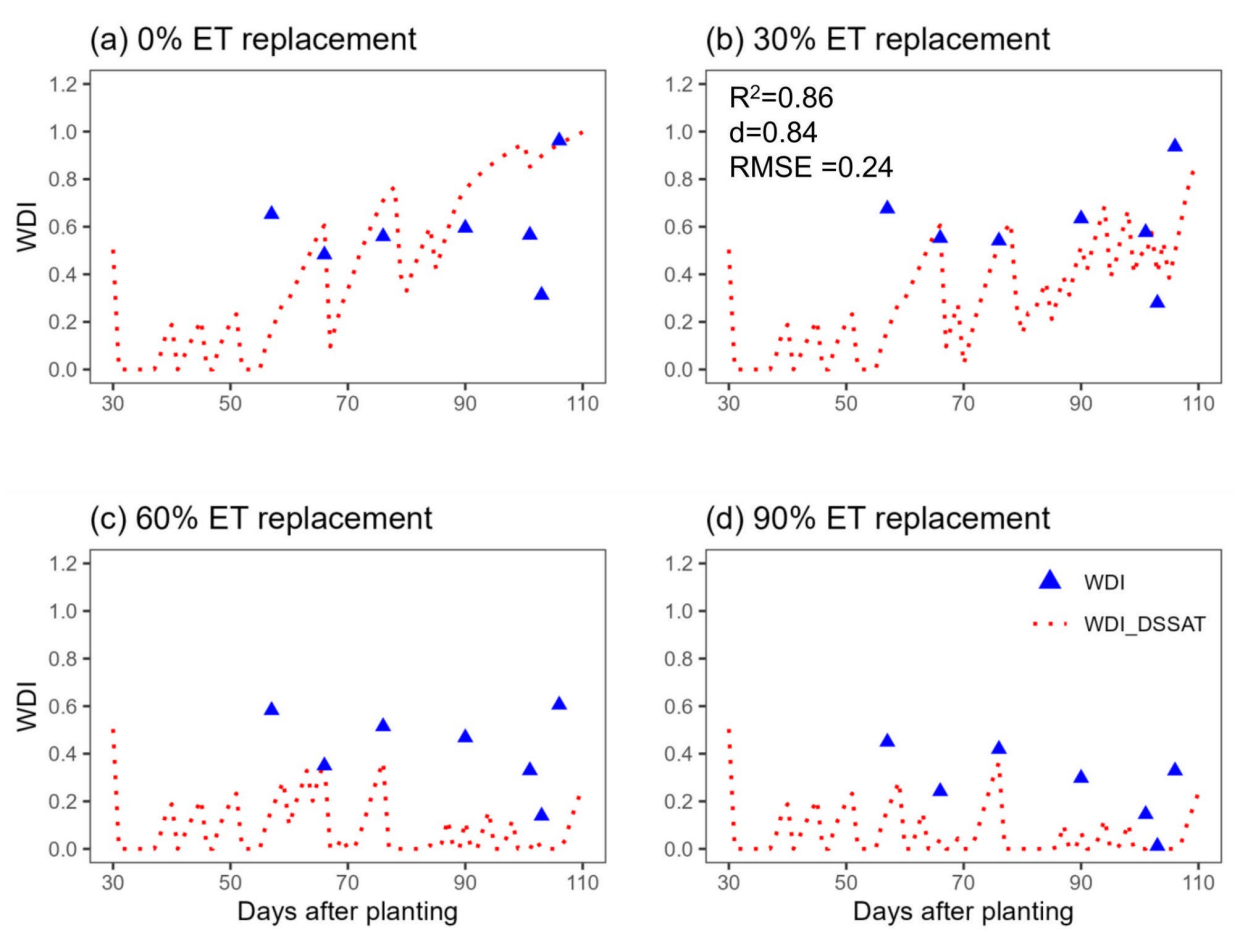
Comparison of Water Deficit Index (WDI) calculated directly using field data from a pivot-mounted sensor package and simulated using the Soil Water Balance method in DSSAT for cotton managed under different ET replacement irrigation regimes in the 2021 growing season.

**Fig. 10:**
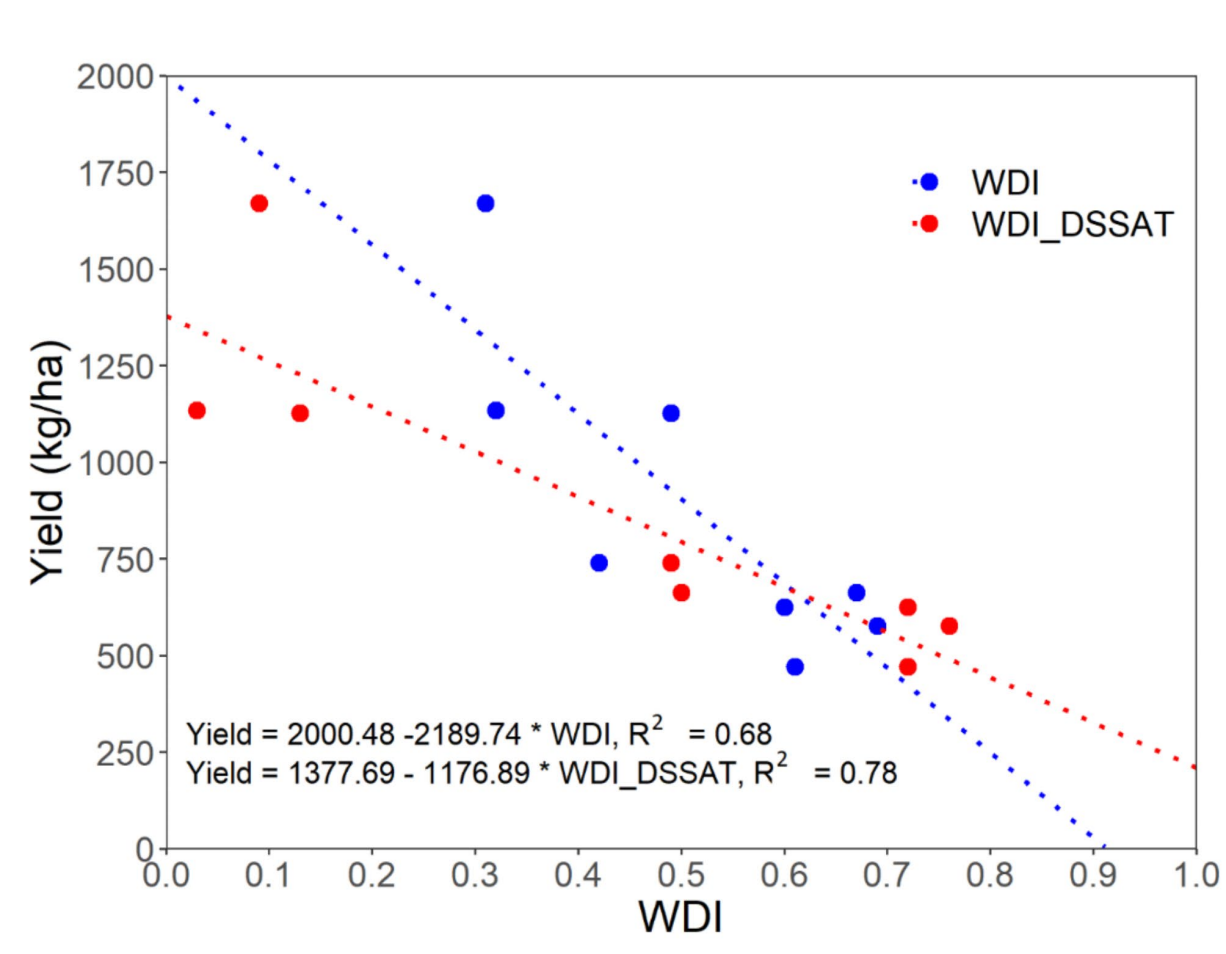
Relationships between seasonal average Water Deficit Index (WDI), calculated directly and simulated using the DSSAT cotton model, and seed cotton yield. Line colors represent the same method as the symbol color (using data of 2020 and 2021 growing season).

**Fig. 11:**
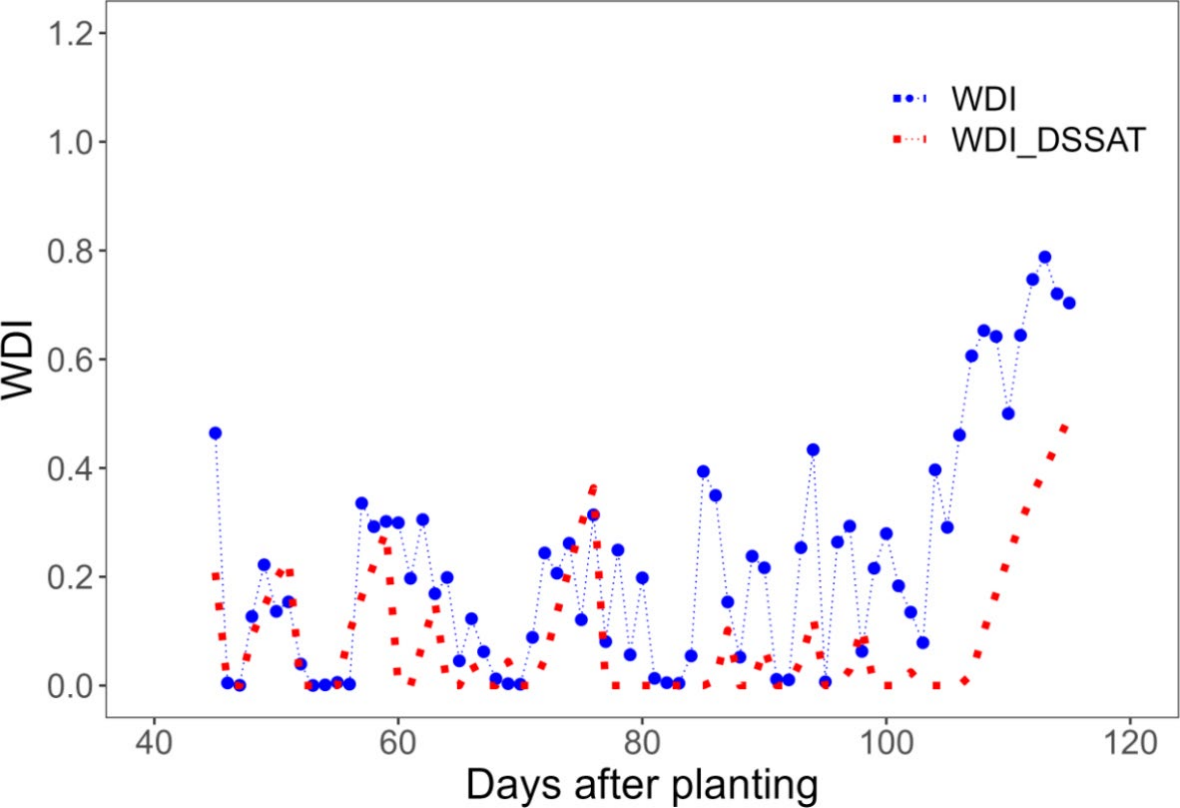
Comparison of Water Deficit Index (WDI) calculated directly using field data from a ground-based station and simulated using the Soil Water Balance method in DSSAT for cotton managed under 90% ET replacement in the 2021 growing season.

Trends in pivot sensor-derived WDI and WDI simulated using the Soil Water Balance approach in the DSSAT cotton model can be seen in Figs. 8 and 9. Although the sensor-based WDI data is sparse, a general agreement of the data with the simulations is apparent, with some exceptions (Figs. 8 and 9). Figure 10 better illustrates those exceptions by presenting the relationships between season-long average sensor-based and simulated WDI with cotton lint yield. The relationship was linear in both cases (R^2^ = 0.68 and 0.78 for sensor and simulation, respectively), but differences in the slope of the relationships suggest that the sensor-based WDI overestimated water stress at the highest irrigation rate. Figure 11 presents WDI results for the ground-based sensing package (placed in a 90% ET replacement plot) versus simulated WDI. There was excellent agreement between WDI derived from two sources, until about 80 days after planting, after which sensor-derived WDI trended higher than simulated WDI. The simulation results indicate the low levels of crop water stress experienced during this period.

### Lint yield

At Chillicothe, cotton lint yield was affected by irrigation in both 2020 and 2021 (Table 2). The 90% ET replacement treatment had the greatest yield, 60% was intermediate, and 30% and dryland yielded the least. There was little (2021) or no (2020) difference in water input, including precipitation and irrigation, between dryland and 30% ET replacement treatments. This was because precipitation limited the amount of irrigation that was applied to the 30% treatment (Table 2; Fig. 5). Irrigation treatments with higher average season-long WDI values had greater loss of lint yield (Fig. 10).

## Discussion

Cotton producers in the U.S. Southern Great Plains region and in many other semi-arid regions of the world need to improve irrigation water-use efficiency and, ultimately, conserve water resources^2,6,7^. Producers typically plan irrigation based on irrigation water availability, affordability of irrigation water, and expected yield^51,52^. IRT- or CWSI-based approaches to irrigation water management have been developed to improve the accuracy and efficiency of irrigation water management, but adoption of these is lacking, likely due to their practical limitations. In the current study, measurements of NDVI, canopy temperature, and weather parameters, each integrated into a pivot irrigation system-mounted package, were useful in calculating WDI as an indicator of crop water stress. Compared to the standard CWSI-based approach that uses a limited number of stationary in-field sensors, this approach allowed daily remote detection of crop water stress right where the pivot was watering, making the data useful to adjust irrigation rates in real time according to crop needs and irrigation goals. With sensors placed on the pivot truss, capture of spatial variability in crop water stress is much better compared to stationary sensors deployed on the ground. Additional IRT and NDVI sensors could be added to the system, installed up or down the line, and averaged to better deal with spatial variability in crop water stress along the length of the pivot. Different aspects of the sensor package and its performance are discussed below.

An estimate of percent canopy coverage or *Fc* is required to calculate WDI. In this study, an approach was taken to estimate *Fc* indirectly through its relationship with NDVI, which can now be continuously measured using modern NDVI sensors. NDVI and *Fc* were measured by UAS from the square formation to boll opening growth stages, then *Fc* was related to NDVI measurements made from the pivot-mounted sensor at Chillicothe (Fig. 2). The relationship between pivot-mounted NDVI and UAS-based *Fc* was linear, but there was considerable variability and an altered slope (R^2^ = 0.77, m = 0.94) relative to purely UAS measurements. Figure 3 shows the relationship between NDVI from UAS and pivot sensors, confirming that the source of the variability was the pivot-derived NDVI. There are at least two possible explanations for the increased variability. First, cotton in this study was planted on straight rows and not on curved path of the pivot (due to challenges in planting on a curve in an irrigation system of such small size). As a result, the pivot-based NDVI sensor “viewed” the crop rows from different perspectives or orientations as it moved across the field, introducing variability. Second, the NDVI-*Fc* relationship would ideally be determined with both measurements made from the same perspective or field of view as the IRT sensor (i.e., at a 45˚ downward angle from the pivot). This could not be accomplished in this study for *Fc*, thus adding another source of variability. Regarding the slope of the NDVI-*Fc* relationship, it is documented in the scientific literature that different sensors and systems used to determine NDVI and *Fc* for cotton output relationships between the two with different slopes^53,54^. In particular, NDVI values can be affected by many factors, including the sensor specifications^55^, plant water stress^56,57^, growth stage^58^, plant nitrogen status^59,60^, as well as soil background reflectance before canopy closure^61,62^.

Although there was a good deal of variability in the in the NDVI-*Fc* relationship as determined in this study, this variability did not seem to be reflected in a similar amount of variability in WDI. To evaluate this, WDI was calculated using both the NDVI-*Fc* relationships illustrated in Fig. 2. In both cases, *Fc* came from the UAS, but the source of NDVI (and variation therein) differed. Figure 6 directly compares WDI calculated using these approaches. The lack of variability in the data suggests that WDI values were not highly sensitive to variability in NDVI. But, as described earlier, both NDVI and *Fc* ideally would have been measured from the perspective of the pivot-mounted IRT sensor to most accurately account for the fractions of canopy and background visible to the IRT. Thus, the WDI values determined in this study may lack accuracy, which is discussed further below.

Another aim of this study was to determine how the source of weather data would affect WDI calculations. This included a comparison of weather parameters (r_s_, Ta, RH, and precipitation) collected by the ground-based mini weather station placed in one 90% ET replacement treatment (standard 2-m height) and the mini weather station mounted on the pivot irrigation system (~ 4 m height). There was generally good agreement between stations: R^2^ between sources for r_s,_ Ta, RH and precipitation were 0.99, 0.98, 0.97, and 0.99, respectively (Table 1). But variability in wind speed measurements between the two stations was somewhat greater (R^2^ = 0.90), despite correction of the pivot wind speed measurements to a 2 m height. It is well-known that wind speed changes with height^35,63^ and weather station anemometers are typically mounted at 2 m to standardize wind speed measurements. Wind speed is inversely related to the aerodynamic resistance, which affects the transfer of heat and water vapor from the crop surface^35,64^. To simulate the effect of changes in wind speed on WDI, the wind speed factor was increased by 10% and 50% in a spreadsheet that was used to make WDI calculations for the present study. This exercise showed that these changes increased the WDI estimates by about 7.7% and 31%, respectively, due to reductions in aerodynamic resistance. The actual variation in wind speed in the current study at 2 m and 4 m height was about 9% and average wind speed was actually greater at 2 m height than at 4 m (4.7 ± 2.0 m s^-1^ vs 4.3 ± 1.8 m s^-1^).

Despite the generally strong agreement in weather parameters between ground- and pivot-mounted weather stations, the season-long relationship between WDI measured on the ground and on the pivot at Chillicothe had a relatively low R^2^ of 0.58 (data not shown). The WDI determined using data from the pivot-mounted sensors generally remained higher for the entire growing season than WDI determined using ground-based data with the IRT sensor directly facing the canopy (Fig. 4). Differences in weather data certainly had some impact, but the strongest contributors to the discrepancy was likely inaccuracy in accounting for the influence of soil background in the pivot-based measurements. As described earlier, *Fc* was determined by UAS from an aerial perspective, rather than from the vantage point of the IRT sensor, which would be expected to lead to some inaccuracy in estimating the lower and upper limits in the WDI model^25,28^.

When comparing WDI derived from the sensor data to simulated WDI, there was not a perfect alignment, but a general agreement in trends (Figs. 8, 9). In sensor-based estimates, especially in the deficit-irrigated and non-irrigated treatments (0% and 30% ET replacement), WDI values experienced sharp decreases with precipitation or irrigation events, while changes in DSSAT-simulated WDI were smoother (Figs. 8, 9). This could be because the DSSAT simulation averaged soil water balance daily, whereas sensor-based estimates were averaged hourly. Perhaps most importantly, the DSSAT WDI simulations were continuous, while the sensor-based WDI estimates were sparce and periodic. When the sensor-derived and simulated WDI results were averaged over the entire season and evaluated for their relationship with lint yield, there was some divergence. The sensor-derived WDI values fit within a relatively narrow range, underestimating plant water stress at the highest irrigation rate (Fig. 10). As discussed earlier, this inaccuracy may be improved by determining the NDVI-Fc relationship from the same perspective as the IRT measurements were made—from the pivot, facing downward at a 45˚ angle. The general alignment of sensor- and DSSAT-based WDI outputs indicates that the sensor package could be used in conjunction with DSSAT to generate irrigation prescriptions for cotton, as described in greater depth by Ale et al.^49^.

In addition to comparing weather parameters collected at different heights at the same location, this study also included a comparison of weather parameters gathered at distant locations. Weather stations are located sporadically throughout the agricultural landscape, with some locations better equipped than others. For example, the Oklahoma Mesonet has weather stations at a higher density than most states or regions of the world^65^. For most cotton producers in the U.S. and worldwide, the closest weather stations may be far from their field sites. Any variation between actual and measured weather parameters will reduce the accuracy of WDI estimates and irrigation prescriptions when relying on off-site weather data. To address this issue, modern, compact, and relatively inexpensive weather stations, as used in this research, could be used to provide on-site weather data. This type of weather station may have somewhat diminished accuracy compared to a standard weather station, but can represent the local weather fairly well. For example, Dombrowski et al.^31^ tested the performance of the ATMOS41 all-in-one weather station and reported underestimation of r_s_ by 3% and ± 7.5% variability in precipitation compared to the standard weather station at the same location. They suggested the higher variability in precipitation was a result of wind-induced errors.

Comparing weather parameters output by the standard weather station at Chillicothe to the mini weather stations on Production Pivots 1 and 2 (within 1.5 to 5 km distance of Chillicothe, respectively), the agreement in measurements of r_s_ (R^2^ = 0.97—0.98), Ta (R^2^ = 0.97—0.98), and RH (R^2^ = 0.94—0.96) were excellent (Table 1). Variability in wind speed was greater among locations (R^2^ = 0.72 – 0.78). But the potential variability was the greatest for measurements of precipitation (R^2^ = 0.46—0.85), as might be expected. Aalbers et al. ^66^ mentioned that the spatial heterogeneity in wind and precipitation, even within small areas, could arise due to inherent chaotic nature of atmospheric/oceanic/land surface processes and their interactions. Weaver and Nigam^67^ reported there is high seasonal and diurnal variability of the wind jet structure and moisture fluxes in the Great Plains region, which is something regional crop producers are well acquainted with.

The WDIs for Production Pivots 1 and 2 were calculated using meteorological data from pivot-mounted mini weather stations integrated into those sensing packages and compared to calculations using data from the standard weather station at Chillicothe (1.5 to 5 km away). There was excellent agreement in WDI using the different sources of weather data, with R^2^ values of 0.95 to 0.96 (Fig. 7). The level of precision in these case studies is likely sufficient that use of an integrated mini weather station or relying on external weather data would likely be acceptable to most cotton producers. Use of a system-integrated weather station would become more important as local weather diverged more from the best external weather source available to a producer.

## Conclusions

A center pivot irrigation system-mounted sensing package with modern IRT and NDVI sensors, and an integrated mini weather station, was found to be effective in distinguishing water stress in cotton among a range of irrigation treatments using the WDI model. Measurements made from production pivots (with LEPA-style emitters) confirmed that the system worked effectively even when the pivot was actively applying irrigation water. Continuous estimates of percent canopy coverage or *Fc* are required in the WDI model. The results showed that output from an angled, pivot-mounted NDVI sensor was linearly related to *Fc* determined aerially by UAS, but with a good deal of variability. The source of the variability was the NDVI measurement and that variability was likely primarily due to the straight (not curved) crop planting orientation in the field, though the WDI output did not seem to be sensitive to the variability. Comparison of pivot- and ground-based WDI indicated that crop water stress was overestimated by the pivot system at the highest irrigation rate. A comparison of the WDI results to crop model simulations of water stress largely showed agreement between measured and predicted values, though the simulations, consistent with the pivot vs. ground WDI comparison, suggested that WDI overestimated water stress at the highest irrigation rate. This inaccuracy may be improved by measuring *Fc* from the same vantage point as the IRT and NDVI sensors (i.e., from the pivot, downward at a 45˚ angle). The general alignment of sensor- and DSSAT-based WDI outputs indicates that the sensor package could be used in conjunction with DSSAT or another model to generate irrigation prescriptions for cotton. Because the sensing system lacks soil moisture sensors or other direct measurements of water, a modeling approach would be required to do this. Integration of a mini weather station into the WDI sensor package effectively represented on-site weather, with generally good agreement observed with a nearby standard weather station. Production fields are usually located far from standard weather stations and, among all measured weather parameters, wind speed and precipitation were particularly improved by using an integrated mini weather station when compared with a distant weather station. Overall, the study confirmed that this approach to cotton water stress detection was feasible, though accuracy could be improved as described above.

## Materials & methods

### Two-year irrigation rate field study

#### Experimental design and site description

A two-year irrigation rate study was conducted at the Texas A&M AgriLife Chillicothe Research Station near Chillicothe, TX, USA (34°11′39’' N, 99°31′07’' W) in the 2020 and 2021 summer growing seasons. A two-tower center pivot irrigation system was used, which was modified with LEPA (low energy precision application) water applicators that emitted water outward at about a 1 m radius from the pivot support trusses. The pivot was equipped with FieldNet (Lindsey Corporation, NE), which allowed irrigation rates to be customized in zones along the pivot path. The irrigation rate was modified by adjusting pivot traveling speed. Half of the area covered by the pivot was utilized for this study, which was divided into 12 pie slices each occupying 1/12th of the area (Fig. 1). The pie slices (plots) were laid out in a randomized complete block design with three replications of four irrigation treatments. The treatments were based on the percent ET replacement method, including 90%, 60%, 30% and 0% ET replacements.

A sensor platform (described fully in the Experimental Procedures subsection) was mounted on the pivot at approximately 10 m proximal of the second (final) pivot tower, as shown in Fig. 1. The span between pivot towers was 46 m (92 m total length from pivot point to final tower), thus the sensors were placed at about 82 m from the central pivot point. This made individual plot lengths (the distance over which the pivot traveled across 1/12th of the field at the position at which the sensor platform was mounted) approximately 21.5 m in length. The plot width was approximately 5 m, corresponding to the field of view of the sensors (Fig. 1).

#### Experimental procedures

All 12 plots were planted with ‘Phytogen 350 W3FE’ cotton (Corteva Agriscience, Indianapolis, IN, USA), a mid-maturing, commercially available cotton variety, on 15 June in 2020 and on 27 May in 2021. Cotton seed was obtained directly from the company. Planting was done with a precision vacuum planter at a seeding rate of 127,450 seeds ha^-1^ and row spacing of 1.02 m with straight rows (i.e., planting across pivot wheel tracks). Fertilizer (60–30-0) was applied after planting in both years at a rate of 140 kg ha^-1^. The weekly irrigation rate for each ET replacement treatment was determined using reference ET output by an on-site standard weather station (Campbell Scientific, Logan, UT) and the published growth stage-specific ET crop-coefficients for cotton^32^. Irrigation was applied in a single weekly dose, except when the irrigation prescription exceeded 25 mm, then the amount was split into two applications, separated by three to four days. In 2020, all plots, including 0% ET replacement, were irrigated on 16 July to wash in applied fertilizer. Fertilizer was washed in by rain on 16 June in 2021. Other management practices followed regional recommendations.

As described earlier and as shown in Fig. 1, a sensor platform was mounted on the pivot at Chillicothe, approximately 10 m proximal from the second pivot tower and 4 m above the ground, to collect in-season data from the field. Crop canopy temperature was measured by an SI-121-SS infrared radiometer (Apogee Instruments, Logan, UT) with an 18° half-angle field of view. The NDVI spectral index was measured by an S2-112-SS spectral sensor pair (Apogee Instruments, Logan, UT) with a field of view of 30° (15° half-angle). The wavelength measurements of the NDVI sensor were 650 ± 5 nm (red) and 810 ± 5 nm (near infrared). The IRT and NDVI sensors were attached to the pivot support truss rod on the forward-moving side of the pivot. The sensors were pointed downward at 45° angle, which allowed measurements to be made without interference from watering operations. At a 4-m height, pointing downward at 45°, and with an 18° half-angle field of view, the field of view of the IRT sensor reached as close as 2.04 m away from the pivot truss. As described earlier, irrigation water application extended outward only about 1 m from the pivot trusses, thus there was no overlap in watering and sensor measurements.

An Atlaslink GNSS smart antenna system (Hemisphere GNSS, AZ) was included to generate sensor platform geolocation information, allowing linkage of pivot and plot locations, and assignment of sensor data to respective plots and treatments. In 2021 (not in 2020), an all-in-one ClimaVue50 mini-weather station (Campbell Scientific, Logan, UT) was added to the sensor platform to collect air temperature, humidity, wind speed and direction, solar radiation, and precipitation data at the exact location of the sensors. It was also placed on the pivot truss rod, but carefully leveled (i.e., not angled downward, like the other sensors). All the instruments were connected to a CR1000X datalogging controller (Campbell Scientific, Logan, UT), powered by a battery and a solar panel, which controlled the sensors and stored the data they collected. The datalogger was programmed to take measurements every five seconds, which were averaged and reported every 30 s.

Sensor data at Chillicothe was collected on dry runs (no irrigation water was flowing) as the pivot was moving (running speed was set at 100%) within two hours of solar noon. Data was collected on 7 July, 6 August, 19 August, and 8 September in 2020, which represented 35, 52, 65, 73 and 85 days after planting. Similarly, data was collected on 22 July, 31 July, 10 August, 24 August, 4 September, and 6 September in 2021, which represented 57, 66, 76, 90, 101 and 103 days after planting. A similar sensor package was also placed on the ground in only one of the 90% ET replacement plots, including an IRT sensor and an all-in-one weather station (no NDVI sensor). The temperature sensor was mounted and adjusted as the crop grew to point downward on the top of the crop canopy. The ground-based system was used as a limited ground truth for the pivot-based WDI measurements, since WDI could be determined on the ground without interference from soil background in the IRT field of view.

An unmanned aircraft system or UAS equipped with a MicaSense RedEdge-MX multispectral sensor (MicaSense, Seattle, WA, USA) was used to collect standard NDVI and percent canopy coverage or *Fc* data. The primary purpose was to generate *Fc* data that could be used to generate a relationship with pivot-based NDVI for input in the WDI model. The UAS sensor had a pixel size of 3.75 μm, resolution of 1280 × 960 pixels, field of view of 47.2 degrees horizontal and 35.4 degrees vertical. The NDVI wavelength measurements of the UAS sensor were 668 ± 10 nm (red) and 842 ± 10 nm (near infrared). The UAS was deployed over the plots on 22 July, 10 August, 24 August, and 6 September 2021 to collect multispectral images, coinciding with the dates when data was collected by the pivot-mounted sensors. All flights were made within two hours of solar noon at 30 m height to collect plot-wide multispectral images. The orange band shown in Fig. 1 indicates the plot area considered in UAS image processing, excluding boundary areas between plots. The imagery was processed using Pix4D Mapper (Pix4D S.A., Prilly) to produce dense point clouds and orthomosaics, which were used for further analysis in ArcMap 10.8.1 (ESRI, Redlands, CA). The zonal statistics tool in ArcMap was used to estimate the average NDVI for each plot. The binary thresholding function in ArcMap, which uses the Otsu method in combination with Zonal statistics tool, was used to estimate the average percent canopy coverage (PCC)^33^. The relationship between pivot-mounted NDVI and UAS-based *Fc* was determined using linear regression.

The plots were mechanically harvested when crop matured using a two-row cotton stripper equipped with an on-board weigh system on 19 November 2020 and 11 November 2021. The samples were ginned to remove trash and seed to determine lint turnout, which was applied to the total plot sample weight to determine lint yield.

#### Determination of the water deficit index

The surface temperature (Ts; canopy and soil), air temperature (Ta), other climatic data, and the NDVI-canopy coverage relationship were used to compute the WDI as described by Moran et al.^28^ and Colaizzi et al.^25^. The surface temperature has theoretical upper and lower limits for a given set of aerodynamic and radiation conditions, which depends on available water for transpiration and evaporation. The measurement of *Ts- Ta* should fall somewhere in between the theoretical upper and lower temperature limits^25^. After estimating the upper and lower limits, the WDI was calculated as follows:

1 $$WDI = \frac{{\left( {Ts - Ta} \right)_{measured} - \left( {Ts - Ta} \right)_{lower\;limit} }}{{\left( {Ts - Ta} \right)_{upper\;limit} - \left( {Ts - Ta} \right)_{lower\;limit} }}$$

where $\left( {Ts - Ta} \right)_{lower limit}$ and $\left( {Ts - Ta} \right)_{upper limit}$ were theoretical estimates for wet and dry conditions, respectively. Assuming that *Ts- Ta* is a linear function of canopy coverage or *Fc*, surface minus air temperatures of a full-cover vegetation surface [$\left( {Ts - Ta} \right)_{vegetation}$], and bare soil surface [$\left( {Ts - Ta} \right)_{bare\;soil}$], the theoretical upper and lower temperature limits can be calculated using Eq. (2) and (3):

2 $$\left( {Ts - Ta} \right)_{lower limit} = Fc \left( { Ts - Ta} \right)_{wet vegetation} + \left( { 1 - Fc} \right)\left( {Ts - Ta} \right)_{wet bare soil}$$

3 $$\left( {Ts - Ta} \right)_{upper limit} = Fc \left( { Ts - Ta} \right)_{dry vegetation} + \left( { 1 - Fc} \right)\left( {Ts - Ta} \right)_{dry bare soil}$$

“Wet vegetation” indicates a well-watered full-cover canopy, and “dry vegetation” indicates a completely water-stressed full-cover canopy. “Wet bare soil” indicates saturated soil, and “dry bare soil” indicates a dry soil surface with no canopy coverage. The relationship between the pivot-mounted NDVI sensor and the canopy cover percentage established in the 2021 season (y = 0.9449x + 0.1539; Fig. 2) was used to compute *Fc* for each observation. To calculate WDI for the ground-based station at Chillicothe, *Fc* was assumed to be 1.0.

Energy balance equations were used to estimate the *Ts- Ta* for wet vegetation, wet bare soil, dry vegetation, and dry bare soil, as described in Colaizzi et al.^25^ and Virlet et al.^34^:

4 $$\left( { Ts - Ta} \right)_{wet\;vegetation} = \frac{{r_{a1} \left( {R_{n1} - G_{1} } \right)}}{{\rho_{a} C_{p} }} *\frac{{\gamma \left( {1 + \frac{{r_{cp} }}{{r_{a1} }}} \right)}}{{{\Delta } + \gamma \left( {1 + \frac{{r_{cp} }}{{r_{a1} }}} \right)}} - \frac{VPD}{{{\Delta } + \gamma \left( {1 + \frac{{r_{cp} }}{{r_{a1} }}} \right)}}$$

5 $$\left( { Ts - Ta} \right)_{dry\;vegetation} = \frac{{r_{a1} \left( {R_{n2} - G_{2} } \right)}}{{\rho_{a} C_{p} }} *\frac{{\gamma \left( {1 + \frac{{r_{cx} }}{{r_{a2} }}} \right)}}{{{\Delta } + \gamma \left( {1 + \frac{{r_{cx} }}{{r_{a2} }}} \right)}} - \frac{VPD}{{{\Delta } + \gamma \left( {1 + \frac{{r_{cx} }}{{r_{a2} }}} \right)}}$$

6 $$\left( { Ts - Ta} \right)_{wet\;bare\;soil} = \frac{{r_{a2} \left( {R_{n3} - G_{3} } \right)}}{{\rho_{a} C_{p} }} *\frac{\gamma }{{{\Delta } + \gamma }} - \frac{VPD}{{{\Delta } + \gamma }}$$

7 $$\left( { Ts - Ta} \right)_{dry\;bare\;soil} = \frac{{r_{a2} \left( {R_{n4} - G_{4} } \right)}}{{\rho_{a} C_{p} }}$$

where *r*_*a*_ = aerodynamic resistance (s m^-1^); *R*_*n*_ = net solar radiation received (W m^-2^); *G* = soil heat flux density (W m^-2^); ρ_ɑ_ = density of dry air (kg m^-3^); *C*_*p*_ = specific heat of dry air (1.013 kJ kg^-1^ °C^-1^); γ = psychrometric constant (kPa °C^-1^); Δ = slope of the saturated vapor pressure–temperature relationship (kPa °C^-1^); *r*_*cp*_ and *r*_*cx*_ = canopy resistance at well-watered canopy and completely-stressed canopy respectively (s m^-1^); and *VPD* = vapor pressure deficit (kPa). The parameters *r*_*a*_*, R*_*n*_, ρ_ɑ_,γ, Δ and *VPD* were computed from equations in the FAO 56 database^35^. To compute *r*_*a1*_, the average height of the cotton canopy was assumed to be 90 cm and *r*_*a2*_was computed for bare soil. To compute *Rn*, surface albedo for the Grandfield fine sandy loam soil (fine-loamy, mixed, superactive, thermic Typic Haplustalfs) at Chillicothe, TX was assumed to be 0.23, 0.23, 0.16 and 0.23 for wet vegetation, dry vegetation, wet bare soil, and dry bare soil respectively, according to Fontes^36^. The values of* r*_*cp*_ and* r*_*cx*_ used in other studies also have varied. Moran et al.^28^ suggested to use *r*_*cp*_ within the range of 25–100 s m^-1^ and *r*_*cx*_ within the range of 1000–1500 s m^-1^ to make the canopy resistance very large or small relative to *r*_*a*.._ Colaizzi et al.^25^ assumed *r*_*cp*_ and *r*_*cx*_ to be 10 and 250 s m^-1^, respectively, for cotton. In this study, *r*_*cp*_ and *r*_*cx*_ values were assumed to be 20 and 650 s m^-1^.

Aerodynamic resistance (*r*_*a*_) was estimated using Eq. 8 from Allen et al.^35^.

8 $$r_{a} = \frac{{{\text{ln}}\left[ {\frac{{Z_{m} - d}}{{Z_{om} }}} \right]{\text{ln}}\left[ {\frac{{Z_{e} - d}}{{Z_{ov} }}} \right]}}{{K^{2} U_{z} }}$$

where, $Z_{m}$ and $Z_{e}$ are the heights for wind and humidity measurements which was equal to 2 m. $d{ } = { }0.67{\text{*average height of canopy }}\left( {h_{c} { }} \right)$ is the zero-plane displacement height at which the wind speed is considered zero. $Z_{om} = 0.123 h_{c}$, is the roughness height governing momentum transfer, $Z_{ov} = 0.0123 h_{c}$ is the roughness height governing heat and vapor transfer. *K* = 0.41 is the von Karman’s constant. *U*_*z*_ is the wind speed (ms^-1^) at 2 m height. For bare soil *d* = 0, *Z*_*om*_ = 0.01 m^37^.

#### Comparison of WDI results with DSSAT model outputs

Sensor-based irrigation scheduling tools can effectively determine irrigation timing, but they do not directly estimate the amount of water to be applied. Therefore, these tools are usually integrated with other methods, such as crop models, to enable real-time irrigation scheduling^38^. For example, the Decision Support System for Agrotechnology Transfer (DSSAT) Cropping System Model (CSM)^39,40^, which simulates water stress based on soil water balance, has been widely used in irrigation scheduling^41–46^.

With proper calibration, DSSAT CSM can be used to simulate crop growth and schedule irrigation by integrating weather, crop genetics, soil properties, and crop management data. The weather data required for model calibration includes daily maximum and minimum temperatures, precipitation, solar radiation, and wind speed, which were obtained from various sources, including DAYMET (1996–2000) (https://daymet.ornl.gov/), the Texas High Plains ET (TXHPET) Network (2000–2012) (Porter et al., 2005), the West Texas Mesonet weather station at Odell, Texas (2013–2017) (http://www.mesonet.ttu.edu/meteograms/), and onsite weather station (2018–2020). Soil data, including soil texture, bulk density, organic carbon content, total nitrogen, cation exchange capacity, pH levels, saturated water content, hydraulic conductivity, field capacity (i.e., drained upper limit in DSSAT), wilting point (i.e., lower limit in DSSAT), and soil root growth factor, were determined based on previous onsite measurement^47^. Information on crop management practices, including planting and harvest dates, tillage, fertilizer and pesticide application, and irrigation, was obtained from practices adopted in the field experiment.

Cotton genotype coefficients were adjusted during model calibration. The DSSAT CROPGRO-Cotton module was previously calibrated and validated for the same study site by Adhikari et al.^47^ using measured data from a cover crop experiment conducted from 2011 to 2015, with an average percent error in seed cotton yield prediction of -10.1% and -1.0% during the calibration and validation periods, respectively. In addition, the simulated soil water in different soil depth profiles (i.e., 0–20 cm, 20–40 cm, 40–60 cm, 60–80 cm, and 80–140 cm) matched well with measured soil water during the calibration and validation periods, with *R*^*2*^ ranging from 0.45 to 0.89 for irrigated cotton fields. Himanshu et al.^48^ further validated the model using data for an extended period from 2011 to 2020. Comparison of simulated and measured soil water content, seed cotton yield, and soil organic carbon content are shown in the Supporting Information (Figures S1 and S2), with calibration details available in Adhikari et al.^47^ and Himanshu et al.^48^.

Ale et al.^49^ further conducted a comprehensive assessment of the calibrated DSSAT model, with an emphasis on its ability to accurately predict seed cotton yield, and irrigation amount and timing across various irrigation strategies. This evaluation employed a 30-year simulation (1991–2020), testing the model over a range of ET replacement treatments (i.e., 0%, 30%, 60%, 90%, and 100% ET replacements). The findings from this extensive evaluation suggest that DSSAT possesses a robust capability to accurately simulate the required irrigation amounts, adapting effectively to diverse weather scenarios and irrigation strategies.

The DSSAT model simulates water stress by analyzing soil water balance, which is directly correlated with the WDI. The water stress coefficient *(K*_*s*_) represents the ratio of actual to potential transpiration rates in crops, varying from 1.0 to 0 in response to the degree of water stress. Under conditions where soil moisture is insufficient, *K*_*s*_ values fall below 1, indicating reduced transpiration due to water stress. In extreme scenarios, when soil moisture is close to the wilting point and plants are unable to extract water, *K*_*s*_ values are close to 0. This dynamic highlights the inverse relationship between WDI and *K*_*s*_. Higher levels of water stress result in lower *K*_*s*_ values, while simultaneously leading to higher WDI values. The relationship between WDI and *K*_*s*_ can be expressed as^50^:

9 $$WDI = 1 - K_{s}$$

*K*_*s*_ can be calculated as^35^:

10 $$K_{s} = \frac{{TAW - D_{r} }}{TAW - RAW}$$

where, *D*_*r*_ is the root zone depletion in mm, *TAW* is the total available soil water in the root zone in mm, and *RAW* is the readily available water in mm. *D*_*r*_, *TAW*, and *RAW* were simulated using a calibrated CROPGRO-Cotton module of the DSSAT model^48^. The top three soil layers in the DSSAT soil file (i.e., 0–5 cm, 5–15 cm, and 15–30 cm) were used for calculation.

#### Statistical analysis

All statistical analyses were run using the SAS 9.4 software (SAS Institute, Cary, NC). Linear regression was used to establish the relationship between *Fc* measured by UAS and NDVI measured from a pivot-mounted NDVI sensor using the PROC REG procedure. The PROC REG procedure was also used to evaluate the relationships and variability of the weather parameters (solar radiation, wind speed, relative humidity, air temperature and precipitation) among different weather stations and among WDI measurements at each site. The data on WDI and lint yield was analyzed by ANOVA using PROC GLIMMIX procedure, keeping block as random effect and irrigation as a fixed effect in the model. Post-hoc means comparisons were made using the Tukey method with a statistical threshold of *P* ≤ 0.05.

The comparisons of the WDI calculated from sensor-based method and those simulated by DSSAT model were made using three model performance statistics, including the coefficient of determination (R^2^), the index of agreement (d-index), and the percent root mean square error (RMSE).

### WDI measurement on production cotton fields

In 2021, two producer-owned and -managed cotton production fields with pivot irrigation systems in Hardman County, TX, USA (Production Pivot 1: 34°12′22’' N, 99°30′59’' W and Production Pivot 2: 34°14′10’' N, 99°30′24’' W) were selected to gather more frequent data from pivot-mounted sensor packages. Production Pivots 1 and 2 were located approximately 1.5 and 5 km, respectively, from the Chillicothe Research Station (i.e., the site of the two-year irrigation rate study). Crop management of these fields was uniform across their respective areas and the approach to sensor data gathering was observational, thus there were no experimental treatments. Production Pivot 1 was a five-tower system (61 m tower span; 305 m total length from pivot point to final tower) and Production Pivot 2 was a seven-tower system (53 m tower span; 373 m total length from pivot point to final tower).

Sensor platforms were deployed on Production Pivots 1 and 2 that included the same components and general setup as the platform used in the two-year study at the Chillicothe Research Station, including mini weather stations, but excluding GNSS systems. The sensor platforms were likewise mounted approximately 10 m proximal of the final pivot towers, with sensors placed on the forward-facing truss rods pointing downward at a 45° angle. The data logging interval was set at 30 min. Like the pivot at Chillicothe, Production Pivots 1 and 2 had modified LEPA emitters, and watering operations did not influence sensor measurements. In the case of these pivots, data was primarily collected while the system was actively irrigating. The data output from the platforms was used to calculate the WDI using the same procedure described above.

All crop management decisions, including irrigation timing and amounts, were made by the cotton producer. Both production fields were planted with the early-mid maturing cotton variety ‘Phytogen 400 W3FE’ on 24 May 2021 with row spacing of 1.02 m. A total of 198 mm and 218 mm of irrigation water was applied over the cotton growing season at Production Pivot 1 and Production Pivot 2, respectively. At both production sites, the pivots were half-pivots (i.e., not full-circle), which limited the amount of sensor data that could be used. The data was filtered to keep only data when the pivots were watering (rotating forward) or were parked on the east side of the fields with the sensors pointed toward the cotton canopy. When the pivots were parked on the west sides of the fields, the sensors were pointed at weeds along fence lines.

## Supplementary information

Supplementary information

## Data Availability

The data that support the findings of this study can be made available from the corresponding author upon request.

## Abbreviations

Tc: Canopy temperature
Ta: Ambient air temperature
Ts: Surface temperature
NDVI: Normalized difference vegetation index
WDI: Water deficit index
Fc: Fraction canopy cover
UAS: Unmanned aircraft systems
