# Supplementary material for: Remote detection of water stress in cotton using a center pivot irrigation system-mounted sensor package
Source: Sci Rep. 2024 Oct 8;14:23436. doi: 10.1038/s41598-024-74092-2 (PMC11461539; doi:10.1038/s41598-024-74092-2)
Supplement: Supplementary file 1 — Supplementary information [file 41598_2024_74092_MOESM1_ESM.pdf]

SUPPLEMENTAL MATERIALS

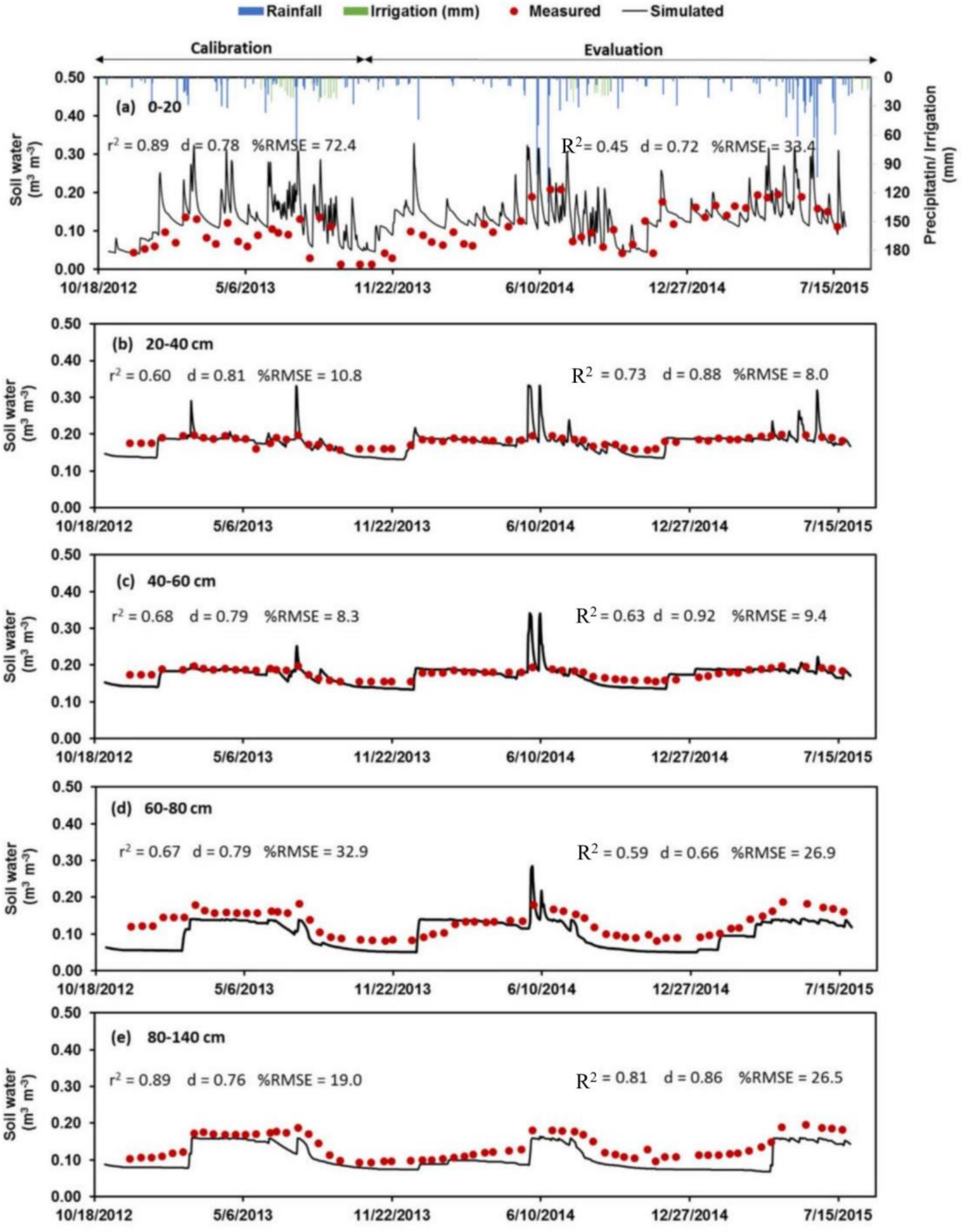

Figure S1: Comparison of simulated and measured soil water content for irrigated cotton without cover crop at Chillicothe, TX. Here, R^2 is the coefficient of determination, d is the index of agreement, and %RMSE is the percent root mean square error. Adapted from Adhikari et al. (2017).

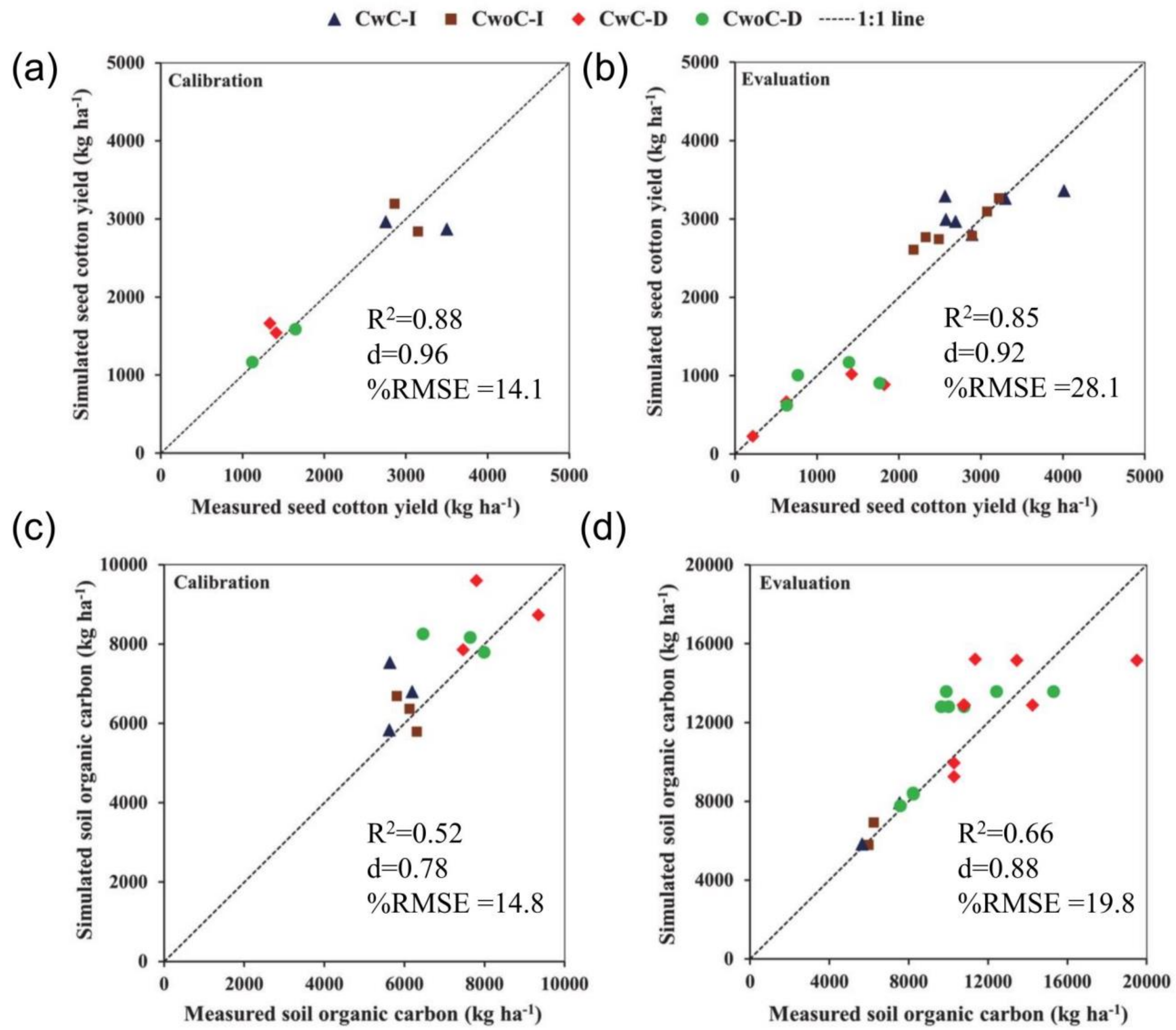

Figure S2: Comparison of simulated and measured seed cotton yield (a, b) and soil organic carbon (c, d) under different treatments at Chillicothe, TX. Here, CwC-I and CwoC-I indicate irrigated cotton with and without cover crop, respectively; and CwC-D and CwoC-D indicate dryland cotton with and without cover crop. R^2 is the coefficient of determination, d is the index of agreement, and %RMSE is the percent root mean square error. Adapted from Himanshu et al. (2022).

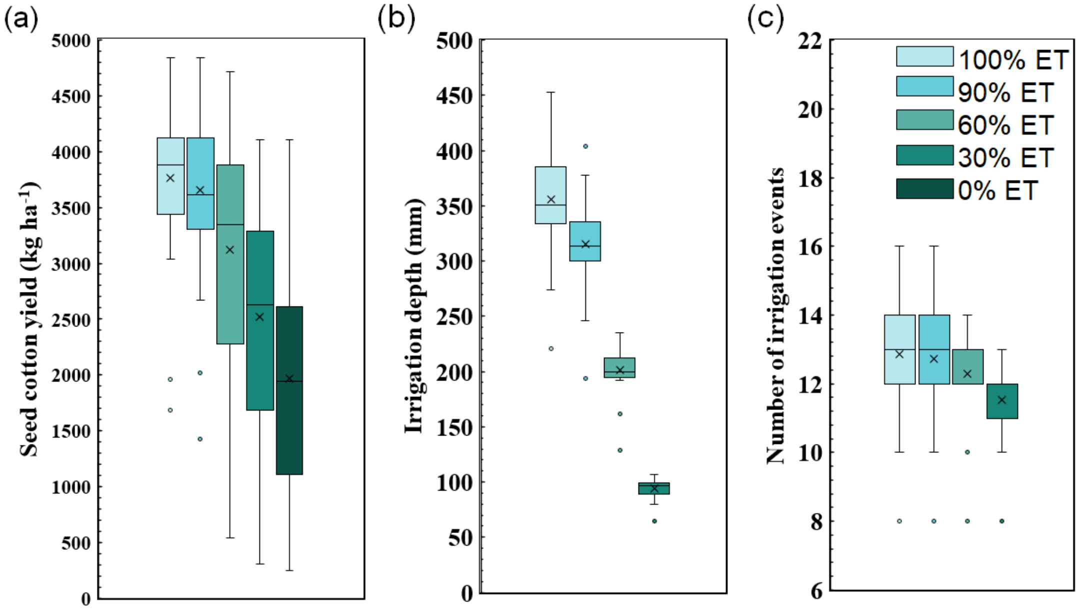

Figure S3: (a) Seed cotton yield; (b) seasonal irrigation depth; and (c) number of irrigation events under different irrigation strategies (1991-2020). Boxplots show the $25^{th,}$ 50th, 75th percentiles, and extremes. Adapted from Ale et al. (2023).
